# Qualitative longitudinal research on the experience of implementing Covid-19 prevention in English schools

**DOI:** 10.1016/j.ssmqr.2023.100257

**Published:** 2023-06

**Authors:** Neisha Sundaram, Nerissa Tilouche, Lucy Cullen, Paniz Hosseini, Patrick Nguipdop-Djomo, Sinéad M. Langan, James R. Hargreaves, Chris Bonell

**Affiliations:** a Department of Public Health, Environments and Society, London School of Hygiene & Tropical Medicine, London, WC1H 9SH, United Kingdom; b Department of Infectious Disease Epidemiology, London School of Hygiene & Tropical Medicine, London, WC1E 7HT, United Kingdom; c Department of Non-communicable Disease Epidemiology, London School of Hygiene & Tropical Medicine, London, WC1E 7HT, United Kingdom

**Keywords:** Covid-19, SARS-CoV-2, Schools, Qualitative longitudinal research, Implementation, Prevention, Preventive measures, England (up to 8)

## Abstract

Implementation studies rarely examine how health interventions are delivered in emergencies. Informed by May's general theory of implementation (GTI), we undertook qualitative longitudinal research to investigate how schools in England implemented Covid-19-prevention measures and how this evolved over the 2020–2021 school year in a rapidly changing epidemiological and policy context. We conducted 74 semi-structured interviews over two time-points with headteachers, teachers, parents and students across eight primary and secondary schools. School leaders rapidly made sense of government guidance despite many challenges. They developed and disseminated prevention plans to staff, parents and students. As defined by GTI, ‘cognitive participation’ and ‘collective action’ to enact handwashing, one-way systems within schools and enhanced cleaning were sustained over time. However, measures such as physical distancing and placing students in separated groups were perceived to conflict with schools' mission to promote student education and wellbeing. Commitment to implement these was initially high during the emergency phase but later fluctuated dependant on perceived risk and local disease epidemiology. They were not considered sustainable in the long term. Adherence to some measures, such as wearing face-coverings, initially considered unworkable, improved as they were routinised. Implementing home-based asymptomatic testing was considered feasible. Formal and informal processes of ‘reflexive monitoring’ by staff informed improvements in intervention workability and implementation. Leaders also developed skills and confidence, deciding on locally appropriate actions, some of which deviated from official guidance. However, over time, accumulating staff burnout and absence eroded school capacity to collectively enact implementation. Qualitative longitudinal research allowed us to understand how implementation in an emergency involved the above emergent processes. GTI was useful in understanding school implementation processes in a pandemic context but may need adaptation to take into account the changing and sometimes contradictory objectives, time-varying factors and feedback loops that can characterise implementation of health interventions in emergencies.

## 1 Introduction

School closures have been widely deployed during the Covid-19 pandemic, resulting in the largest disruption to schooling in history, affecting 1.6 billion students in over 190 countries (UNESCO, 2021). School reopening was accompanied in most countries by implementation of various preventive measures (Guthrie et al., 2020; Hoffman et al., 2021; Krishnaratne et al., 2020; Melnick & Darling-Hammond, 2020; Sundaram et al., 2021; UNESCO, UNICEF the World Bank & OECD, 2021). In England, an estimated 9 million students were affected by school closures (School Census Statistics Team, 2021) when schools were closed to all except vulnerable or key workers’ children. After a national lockdown, primary and secondary schools reopened to all students in September 2020. Department for Education (DfE) prevention guidance established a system of controls that schools were expected to implement following risk assessments (Department for Education, 2020a). These included requirements for: unwell individuals staying home; use of face-coverings in communal areas; hand and respiratory hygiene; enhanced cleaning; active outbreak-management; minimising contacts by keeping individuals in groups (“bubbles”); and physical distancing (Department for Education, 2020a). Schools were again closed to all except vulnerable students and the children of key-workers from 5 January to 6 March 2021 (Cabinet Office - Government of the United Kingdom, 2021). When schools reopened, additional measures were emphasised including: ventilation; regular asymptomatic testing; and use of face-coverings in classrooms for secondary-school students (Department for Education, 2021). Reopening with preventive measures in place has required immense efforts, adjustment and accommodation by school leaders, staff, students and parents/carers (Andrew et al., 2020; Branquinho et al., 2020; Kim & Asbury, 2020; Kim et al., 2021; Lundie & Law, 2020; Education Support, 2020).

Existing studies published to date focusing on implementation of Covid prevention in schools have suggested that, in the main, schools implemented a range of preventive measures with generally good fidelity, albeit with variation between measures and between school types, and that these have largely been acceptable to staff, parents and students (Guthrie et al., 2020; Hoffman et al., 2021; Hommes et al., 2021; Lorenc et al., 2020; Sundaram et al., 2021). Valuable as these ‘snapshot’ studies have been, little is known about the social processes underlying implementation, and how these evolved over time and varied in relation to epidemiological and policy context.

Examining implementation of preventive measures using qualitative longitudinal research (QLR) and informed by May's general theory of implementation (GTI) provides an opportunity for understanding how implementation processes evolve over time, how they vary with epidemiological and policy context and how they come to be sustained in the long term. GTI provides a suitable theoretical framework for such research and proposes that complex interventions come to be enacted by local agents through processes of: (i) ‘sense-making’ (understanding the intervention); (ii) ‘cognitive participation’ (committing to delivery); (iii) ‘collective action’ (collaborating with others in implementation), and (iv) ‘reflexive monitoring’ (assessing the success of implementation and determining further actions) (May 2013). GTI proposes that these processes are influenced by an intervention's ‘capability’ (whether it is workable and can be integrated within a social system), local ‘capacity’ (whether there are supportive roles, norms and resources present in a setting) and potential (whether local agents hold positive attitudes to the intervention).

Using GTI as a framework to examine Covid-19 preventive measures in schools presents a novel opportunity to explore the challenges to and facilitators of implementation in an emergency and how well GTI provides a framework for understanding these. Covid-19 prevention provides an interesting case-study for the application of this theory because implementation involved an emergency context, which is characterised by rapidly changing epidemiology and policy, and multiple measures, many in interaction with each other, which changed over time.

Informed by QLR conducted through the 2020–2021 academic year and using GTI as a framework, this paper aims to examine how a sample of schools participating in a longitudinal research study in England implemented Covid-19 prevention measures in an emergency and how this changed over time through the accounts of staff, students and parents. It asks what processes were involved in implementing these measures and how were they influenced by school, policy and epidemiological context.

## 2 Methods

### 2.1 Study design and setting

This QLR study was nested within England's national Covid-19 Schools Infection Survey (SIS), an observational cohort study conducted in state primary and secondary schools across 15 selected local authorities (LAs) in England differing according to incidence of Covid-19 and tracking these through the 2020/2021 school year. Further details of the overall SIS study are reported elsewhere (Halliday et al., 2022; Hargreaves et al., 2022).

The present study involved two rounds of interviews with headteachers, teachers, parents and students. With a view to conducting the QLR in at least six English schools, we selected eight to allow for potential drop-outs. We first contacted all schools enrolled in the SIS study to determine their interest in participating in interviews. From interested schools, eight were purposively selected to reflect diversity in: initial reports of extent of implementation of preventive measures from SIS surveys; local deprivation (income deprivation of students’ families as indicated by the proportion of students eligible for free school meals); and school type (primary/secondary). Of the eight, one school did not engage other than an initial headteacher interview and repeat interviews with one teacher.

### 2.2 Participant selection

School-engagement officers within the SIS study contacted the eight schools introducing researchers to headteachers. We first interviewed each school's headteacher and requested headteachers share our details with two other staff-members, two parents and (at secondary schools only) two students who might have been willing to be interviewed. We aimed to ensure purposive diversity of participants based on seniority/role for staff-members and gender for parents and students by explaining this requirement to the headteacher. At the end of the first interview, participants were asked if they could be contacted for a second interview the following term. For these second interviews, the research team re-contacted participants via emails and phone-calls.

### 2.3 Instruments

Semi-structured interview guides focused on: experiences at school during the pandemic; perception and adoption of preventive measures; challenges and facilitators of implementing preventive measures; and wellbeing. Interview guides were informed by previous research conducted in summer 2020 (Sundaram et al., 2021), publicly available government guidelines on school prevention and discussion with experts.

### 2.4 Data collection

In spring and summer terms 2021, telephone interviews were conducted by experienced social science researchers (NS, NT, LC and PH) by telephone. Study information and consent forms were sent in advance and written consent was sought from all participants. As written consent was not always provided, verbal consent was obtained from all participants before the interview and documented. Interviews were audio-recorded with participants’ permission and lasted 30–75 ​min. No financial compensation was provided.

### 2.5 Data management and analysis

Interviews were transcribed verbatim from audio-recordings and anonymised. Where participants did not wish to be audio-recorded, interviewers made notes. Transcripts and notes were managed and analysed using MAXQDA 12 (VERBI GmbH).

GTI (May, 2013) informed the interview guides, and guided analysis and interpretation. The analysis involved an iterative process moving between theory and empirical insights using abductive logic and reasoning with a focus on time. As abduction allows for ongoing refinement and emergence of processual insights, it is considered an ideal fit for the dynamic and cumulative nature of QLR (Neale, 2021). Analysis was done by NS and reviewed by CB. For each round of interviews, data were coded thematically (Braun and Clarke, 2006) based on initial codes informed by the interview guide and GTI. Preliminary codes were then refined and new themes were identified inductively. Initial and follow-up interviews for each individual were then analysed together as a unit to explore temporal dimensions. Data from headteachers, teachers, parents and students were compared and contrasted within each theme.

### 2.6 Ethics

This study was approved by research ethics committees at the UK Health Security Agency (ref: NR0237) and the London School of Hygiene and Tropical Medicine (ref: 22657).

## 3 Results

Across four primary and four secondary schools, 43 interviews were conducted in a first round between February–April 2021 (spring term) and 31 repeat interviews were conducted between June–July 2021 (summer term) (Table 1). All but one participant in each round consented to audio-recording. Primary schools in our study had between 220 and 650 students and secondary schools had 520-1310 students. Rates of free-school-meal eligibility, an indicator of financial deprivation, ranged from 11% to 30% in primary, and 7%–58% in secondary schools.

**Table 1:** Summary description of schools and participants.

	Primary schools (4)	Secondary schools (4)
*Geographical region∗*		
North West	1	2
North East	2	2
South East	1	0
Boys/Girls∗	All mixed	All mixed
Total number of students (range, rounded to nearest ten)∗	220–650	520–1310
*Ofsted Rating∼*^*,*^^a^		
Outstanding	0	1
Good	4	1
Requires Improvement	0	1
Not available	0	1
Percentage of pupils on free school meals (range)∗^,^^b^	10.8–30.3%	6.9–57.7%
**Number of interviews in Spring term**^c^		
*Headteacher*	4	4
*Teacher/Teaching assistant*	8	8
*Parent*	7	6
*Student*^d^	n/a	6
**Total**	**19**	**24**
**Number of interviews in Summer term**		
*Headteacher*	3	4
*Teacher/Teaching assistant*	8	4
*Parent*	6	5
*Student*^d^	n/a	1
**Total**	**17**	**14**

∗ Source: https://get-information-schools.service.gov.uk/Search. Accessed on 29/07/2022;

∼ Source: https://www.compare-school-performance.service.gov.uk/. Accessed on 29/07/2022;

a Ofsted: Office for Standards in Education, Children's Services and Skills, is the UK government department responsible for inspecting schools. Rating is on a four point scale: Outstanding, Good, Requires Improvement, Inadequate. One school is n/a as it is a new academy and not been rated yet.

b This shows the percentage of students eligible to receive a free school meal. Students or their parents can claim free school meals if their household income is below a certain threshold or they received qualifying benefits from the government. It may be considered an indicator for income deprivation.

c Participant gender (as determined by researchers during the interview, as this was not explicitly asked to participants) was as follows: Headteachers – 3 female, 5 male; Teacher – 16 female, 0 male; Parent – 11 female, 2 male; Student – 2 female, 4 male.

d Primary-school students were not included in the study; only secondary-school students were interviewed.

In round 1 (spring term), all participants discussed experiences of the autumn 2020 term with participants interviewed after 8 March 2021, when school re-opened to all students (n ​= ​24), also discussing the spring 2021 term. Round 2 focused on experiences during the summer 2021 term. The following themes are structured in terms of GTI constructs.

### 3.1 Sense-making

Given most participants’ limited previous experience of epidemic control and the emergency context, there was an initial phase of urgent and rapid sense-making, followed by gradual consolidation over time. Headteachers, supported by their senior-leadership teams, were primarily responsible for translating guidance received into concrete school plans. These were then disseminated to be implemented by teachers, students and parents. Sense-making varied between groups as discussed below.

#### 3.1.1 Headteachers and school leadership teams

Schools were required to implement DfE guidance to reduce risk of transmission of infection. Headteachers and school leaders found DfE guidance to be a useful starting point but it also presented challenges for sense-making, with challenges centring on intervention ‘capability’ (whether it was workable and could be integrated within a school). First, DfE guidance was reported as issued at short notice, which meant school staff had to work over the holidays with little time to interpret and discuss the guidance. Second, interpretation was hindered by lengthy documents perceived by headteachers as not user-friendly and lacking practical examples to help understand what exactly was required. Participants reported that subsequent updates did not clearly indicate which sections had been revised, complicating the process of understanding what was needed.

“If you want something that makes you feel reassured that you’re following procedures that would be appropriate ones, it’s good in that sense. But they’re cumbersome” (Secondary, Headteacher)

“Some of it is straightforward, but the issue is that a lot of people who work in DfE don't work in schools … It's not necessarily that the content is difficult to understand, it's that the translation of the content into reality in a school is sometimes difficult to implement. So it's not necessarily the understanding of what the goals are … It's about how that actually is articulated into action within schools” (Secondary, Headteacher)

Opinion varied as to whether there had been improvement in DfE guidance since summer 2020, with some noting minor improvements. For most, concerns remained around the challenges arising from receiving last-minute updates and the lack of clarity, which impacted planning and school functioning.

Sense-making was also influenced by institutional ‘capacity’ (whether there are supportive roles, norms and resources present in a setting). Headteachers and senior leaders responsible for interpreting the guidance and developing plans for implementing preventive measures found this a time-consuming task as it was done in addition to other responsibilities. Schools which were part of a multi-school trust, and thus had additional support for interpreting guidance and developing risk assessments, found this extremely helpful as it took some pressure off the headteachers.

“Because we’re part of a trust, we do have the operations manager, an estates manager who have looked at in detail, and then [produced] the risk-assessment and then they will be able to support the operational check-list. So I think it would have been more difficult if it was just me as a standalone headteacher.” (Secondary, Headteacher)

Another example where sense-making was influenced by ‘capacity’ related to additional support promised by the DfE to help schools navigate management of Covid-19 infections by providing personal advice over a telephone helpline. One school found this useful:

“I know some headteachers have reported negative feedback, but the DfE helpline has been really helpful. The guidance is very clear as to what you do.” (Primary, Headteacher)

But other headteachers considered it a wasted opportunity and not as helpful as it could have been.

“The DFE helpline was a bit of a misnomer: it wasn’t much help. All they basically did was tell us what we already knew and then said ‘we’ll pass it onto public health’. But we were told we had to contact them every time and after the fifth time they said ‘only contact us every five times’. I think they weren’t expecting to have the increasing cases at the time and so they became less involved. So they told us not to ring them after about two or three weeks. They said there was no point contacting them, just to go through our local systems.” (Secondary, Headteacher)

School leaders were keen to make sense of what needed to be implemented in their schools, as they felt responsible for keeping schools as safe as possible and to abide by legal requirements. In that sense, the local ‘potential’ (whether local agents are positive about enacting an intervention) for the intervention was very high. However, discontent about how guidance was communicated to schools by government authorities (including the perception that it was sometimes leaked to the media before it was provided to schools) and perceived inadequate support received in operationalising the guidance led to an erosion of trust in these authorities over time. One headteacher, for example, perceived a lack of support and understanding of teachers' efforts by those issuing guidance:

“The DfE are just shocking … I feel like they’re sending school teachers just out to the wolves. I hate that. I’m so sorry, I hate that … They make me upset … They sit in their offices, or they sit down in, in Westminster making up these rules. They might come out to a couple of schools and they might see some Covid bubbles. They just don’t have a clue …” (Secondary, Headteacher)

Sense-making was further complicated by a diversity of sources of information, which, in addition to government, included trade unions, local authorities and academy trusts. This was sometimes contradictory. A headteacher reported having to adhere to guidance from government alongside requests from teachers’ unions, which often did not align.

“Currently the [union] are saying that they would like all teachers to be vaccinated before they return to school and that they would like fewer children in the class. They would like, you know, the metre distances between the tables, etc. which is not the same as the government guidance. So I think difficulties in terms of leadership is trying to match up the requirements. Especially since there’s members of staff who are anxious because those requirements are not being met.” (Primary, Headteacher)

However, diverse information sources could also be useful in the absence of clear and practical guidance from the government. Many headteachers reported relying on guidance from their local authorities, local public-health teams or headteacher networks, which were considered useful in understanding the local situation and therefore highly valued.

“Everyone was kind of making it up as they go along. I know that for a fact because we'd have conversations with other headteachers via the Head Teacher Association, and we’re asking each other ‘What are you doing? Well, I'm doing this, what are you doing?’ And then we'd have conversations with our union reps, and our union members. None of us, none of us virologists, we’re teachers with teaching degrees … I'm not an expert in infection control.” (Secondary, Headteacher)

Over time, school leaders increasingly developed their own plans that drew from and processed multiple information sources in addition to government guidance, which were disseminated to school stakeholders. There was a steady growth in confidence and ‘capacity’ for decision-making among headteachers. As explained by a secondary-school headteacher, this allowed their school to *“react to local circumstances ahead of national announcements”*. (Secondary, Headteacher, 2nd interview)

“Where at first … I did need the support from Public Health England to question and check the data, [to now] where I feel that I could be a Public Health England adviser myself. At first I was relying on them for the data and things like that, but now I know.” (Secondary, Headteacher)

#### 3.1.2 Teachers

Once plans were made by leaders, these were disseminated to staff-members for implementation. This was generally done through emails, weekly online meetings and “open-door” online meetings for teachers to drop into. One school built-in staff assurance time before the students returned. This additional consideration given to building ‘capacity’ was believed to have improved sense-making for teachers. A primary headteacher commented on the challenge of securing staff engagement when teachers were receiving information from multiple sources:

“From a headteacher’s point of view, we’ve got to manage the fact that they’re getting messages from the news, messages from the unions and they’ve got anecdotal information through social media. All of those cause anxieties and worries, whether they are falsehoods or whether they are the truth, it doesn’t really matter. And then therefore what you’ve got is not just the facts about school or how it’s going to run but that increased anxiety for each individual in a different way and how they manage it.” (Primary, Headteacher)

Teachers repeatedly emphasised the importance of open communication and sharing information on the rationale for processes being implemented. This was considered essential for understanding what was required, building trust and allaying concerns. A majority of teachers interviewed felt informed through honest and regular communication from headteachers, having the opportunity to ask questions and provide feedback and trusting senior leadership. However, some felt they did not have a chance to express their thoughts on the measures being implemented, or that their individual challenges with COVID-19 were not understood. Where leaders were perceived to have held back information, this could make teachers anxious. The ‘potential’ for sense-making among teachers was therefore influenced by their trust in leaders and the guidance provided by their schools over other, disparate sources of information. School leaders' willingness to listen to and understand challenges being faced by teachers could influence teachers' trust in decisions made by leadership regarding implementation.

“When we’ve had staff briefings, everyone can contribute to ideas, everything is run by us before it’s finalised. So it’s not like a senior member of the team will make a decision and then you’ll have to follow. I think it’s quite collaborative how we’ve been working and makes sure everyone’s opinions are heard. “ (Primary, Teaching assistant, 2^nd^ interview)

#### 3.1.3 Students and parents

Schools invested in the process of sense-making for their students and parents. Students mentioned being informed of new measures primarily through email, while some noted their school's use of popular channels such as Twitter and YouTube. Among students interviewed, all found the information provided by their school easy to follow and understood the need to have preventive measures in place.

“*I do feel like school’s covered it quite well and they’ve made sure that all the advice is quite clear.” (Secondary, Student)*

Open communication was deemed important for sense-making for parents too and helped reassure them. Some noted innovative means of communication including visual colour-coded blueprints to explain areas designated for each year-group, and all parents we spoke with were happy with the communications received.

“*There’s been a lot of open conversations. There’s been a lot of information sent from school, you know emails or texts of whatever. They’re very open about why they’re doing things, so the decisions that they’ve come to. They’ve been amazing.” (Primary, Parent, 2*^*nd*^*interview)*

A primary school with a high proportion of students whose parents spoke first languages other than English emphasised the importance of finding ways to communicate in parents’ first language, or communicating via video which is easier to follow and allows for use of gestures.

### 3.2 Cognitive participation

COVID-19 prevention involved various measures. Schools and teachers strongly committed to implementing many of these to minimise transmission, reassure colleagues, parents and students, and abide by government requirements. Many measures, such as enhanced cleaning, one-way systems (where unidirectional movement of individuals is instated to minimise face-to-face contact between persons in the school premises) and frequent handwashing, garnered cognitive participation that was sustained over time.

Some measures, however, did not garner cognitive participation in some schools where there were doubts about their ‘capability’ (workability). For example, distancing between students in the classroom was not committed to in some schools because it was not considered feasible due to limitations in classroom size. This was mostly the case for secondary schools where such distancing was required. This was explained by a headteacher as follows:

“Your classroom is 10 metres by 12 metres. I can’t physically move the walls. I’ve still got 30 kids in the classroom. So it is not feasible to be able to space children in a classroom. You can have them all sat in one direction, so that would be not facing each other. But you cannot change the physical space of a school.” (Secondary, Headteacher)

Similarly, adequate ventilation in the classroom was not considered possible in some schools where windows could be opened slightly or not at all. These schools reported not receiving additional support to improve ventilation. A headteacher explained:

“The ventilation is only a window that opens about three or four inches, so the ventilation isn’t great.” (Secondary, Headteacher, 2^nd^ interview)

Through our QLR, we identified that cognitive participation varied with epidemiological context. Initially, when schools were closed to a majority, many schools committed to distancing measures with the knowledge that it was better than the alternative of even more stringent measures or school closure.

“I don’t have an issue with putting new strategies in place because it hasn’t been, you know, the teacher hasn’t been coming in with a hazmat suit on. You know, those kind of things must be worrying. But if the teacher just looks normal, generally acts normally, although is keeping a distance, that in itself is good.” (Primary, Headteacher)

However, over time, in some schools, some interventions came to be regarded as unworkable. This mostly related to measures that were perceived as undermining schools' mission to promote student education, development and well-being. For example, some teachers were less prepared to distance themselves from students who needed help as the immediate emergency context dissipated. During periods of relatively low Covid-19 prevalence, many participants were less accepting of the trade-off between preventive measures and students’ learning and well-being. Teachers reported staying in front of the classroom as far as possible, but over time used their discretion in deciding when to support students at close quarters. In many cases, staff employed other preventive measures, such as face-coverings, to manage risks in such circumstances.

“Yes, [distancing] is possible but it’s not great teaching and learning. So, I can have my members of staff at the front of the classroom, and say ‘you’re not allowed to go past this line, because that puts you in a danger zone’. But if I want to go and see what a child has written in her book, or go and help a child struggling, I’ve got to risk-assess it. So even when I was doing like covering lessons, because we had staff off sick, I put my mask on and I moved to where the child was, and I’d have to go and have a look, because that's the right thing to do is to make sure that child is okay.” (Secondary, Headteacher)

Similarly, keeping students in “bubbles” proved challenging in secondary schools as some mixing was considered unavoidable. It was also not considered sustainable because it contributed to increases in staff workloads. Keeping students in bubbles was considered workable in primary schools and consistently implemented, but not without challenges. A primary-school parent who had previously described bubbles as the best prevention measure in place, subsequently commented on their not being a long-term solution during the second interview, as it felt unjust to students whose “bubbles” had been sent home many times compared to those who had had minimal disruption:

“I just don’t think the bubbles are sustainable, because some children have been unfairly hit by it.” (Primary, Parent, 2^nd^ interview)

A primary school that strictly maintained bubbles reported that keeping students in bubbles meant they could not provide speech and language interventions to students, and also could not use the lunch hall. This school and others also noted their aspiration for normality despite their commitment to these measures.

“I think it will be nice to get to a place where the children are able to mix a little bit more … I think moving forward it’s [reference to bubbles] still fine, but it will be nice to be able to get to normality.” (Primary, Headteacher)

Students and parents aspired to normality too, and for participants in our study, this meant being able to attend school. Secondary-school students noted their commitment to the measures as they were keen on keeping schools safe so they could stay open. A student reflected that most of their classmates committed to the preventive measures advised, as they did get easier over time.

“Social distancing can sometimes be a bit [challenging] just because you’re not really used to it. But then when you actually think about it, it’s quite easy to do.” (Secondary, Student)

### 3.3 Collective action

Implementing preventive measures in school required collective action involving many stakeholders: staff, students, parents/carers, local authorities, public-health agencies and the DfE. Primary and secondary headteachers and teachers thought that students and staff generally adapted well to new routines and preventive behaviours. Students echoed this sentiment. One teacher noted:

“The kids are fantastic. They really are good. Even in the year two class, who are really quite a challenging class, we come in, they automatically just start handwashing while you're teaching. That's five, six year olds … It's very calm, and if an adult forgets, the kids will remind them.” (Primary, Teacher, 2^nd^ interview)

Similarly, students and parents were appreciative of teachers' and headteachers' efforts. A student said of their school in both the first and repeat interviews that *“they've done everything they can” (Secondary, Student, 2*nd *interview*).

A parent of two children echoed this sentiment emphasising the sense of safety these efforts afforded:

“I've only got praise really for our school. The measures that they took, the things that they've done, the preparations that they did for September, the feeling of safety, my kids feeling safe, the teachers putting every effort into everything. Yes, I just feel the school has been just such a positive environment throughout all of this.” (Primary, Parent)

One secondary headteacher emphasised behavioural adaptions to the new system by creating routines in school, training staff and students to follow these routines and reinforcing positive behaviours until they were routinised, as an effective means to collective action. Similarly, staff-members also came together to enact preventive measures and guide students in doing so, and the ‘potential’ for collective action was high.

“They understand the routines and expectations in school are so ingrained it is going to be hard to get out of them again I think, rather than whether the staff are following them. The staff are wearing their face coverings all the time in shared areas, way more than I thought we would.” (Primary, Headteacher, 2^nd^ interview)

At the same time, a headteacher noted that some staff, especially those who felt more vulnerable to Covid-19, found it difficult to adapt to changing guidance and loosening of restrictions. This could hinder collective action within the school and increase pressure on the headteacher:

“I do have a group of staff who were reluctant to move around bubbles. Although the guidance from the DfE says we can, they, you know keeping distance, they are reluctant to put themselves within that process and that risk. And I can’t ask them to start to do that. I don’t feel it’s my place to do that. So therefore the only person who can move around bubbles is me. So that’s where I cover a lot.” (Primary, Headteacher, 2^nd^ interview)

Adherence to some measures, such as handwashing and face-coverings, improved with time as these were routinised and became embedded into everyday practice.

“I think the fact is everybody got used to it. So little things like we sanitise our hands when we leave or enter a room. When you go and sit in a room with other people, you don’t go and sit next to them. I think people are used to it by now.” (Primary, Headteacher, 2^nd^ interview)

“Yeah, it was tough at first because it was totally new, but it became easier, so masks, hand sanitising, washing desks before and after.” (Secondary, Student)

Wearing face-coverings was initially considered unworkable by some secondary headteachers, but over time schools' ‘capacity’ to organise collectively to implement this improved. Student ‘potential’ to wear face-coverings varied across different ages and a headteacher reported that younger secondary-school students were often more willing to adhere to wearing face-coverings than were older students. Primary school students were not required to wear facemasks as per DfE guidance. From a student perspective, face-masks were more likely to be used when they were clearly prioritised:

“In October, November, December time we were sort of told to wear masks but it wasn’t really enforced, so back then not many people wore the masks. But then these past three weeks everyone’s really been putting an effort to put the masks on, and there haven’t been as many cases.” (Secondary, Student)

However, headteachers also noted that, over time, familiarity with some measures resulted in reduced vigilance, especially when infection rates in the community and perceived risk were declining.

“I’d say in the first half of summer, when life in school felt a little more normal, because trips started happening and visitors, and we didn't have any bubbles close. But we have to make more of a conscious effort to remind people, you know, in the staff room to make sure you’re still socially distanced … It's very easy to forget that it's still out there.” (Primary, Headteacher, 2^nd^ interview)

This decline in ‘potential’ was mainly noted with regard to parents, particularly during the second round of interviews. A primary-school headteacher noted with frustration in a second interview that, despite a high number of Covid-19 cases, some parents weren't following government or school rules and were, for example, going on holiday when they were meant to be self-isolating, or not wearing face-coverings. It was felt that parents could be reminded to follow the rules, but they could not be enforced. Headteachers also lamented lack of government support in requiring some measures, such as face-coverings, for parents.

“We have encouraged parents to wear face coverings. Not all of them do but you can only encourage it, you can’t insist on it.” (Primary, Headteacher)

“The other issue is the government have absolutely, categorically not stated that parents should be wearing masks on the school ground … we didn’t get enough back-up for that.” (Primary, Headteacher)

Asymptomatic testing with lateral flow tests (LFTs) was recommended in March 2021 when schools reopened to all students. The guidance required secondary-school students be offered three tests on-site at school in the first week, followed by home-testing twice-weekly thereafter. At primary schools home-testing was required only for staff, not students. Secondary-school headteachers found implementing on-site testing extremely challenging as they lacked staff (‘capacity’) to administer and manage these processes and had concerns about bringing potentially infectious students into schools to conduct the test. One headteacher explained the capacity challenges and frustration with government guidance for on-site testing as follows:

“How can I, how can I test children when I've got 20 staff out of the building? I’m just barely keeping an adult in front of a class and they want me to do mass testing?” (Secondary, Headteacher, 2^nd^ interview)

In contrast, primary-school headteachers reported positive experiences implementing home-based LFTs distributed at school, considering it beneficial in reassuring staff of their safety:

“I think LFTs are pretty straightforward, they’re easy to manage. It helps everyone else feel safe. I think they’ve been a good preventative measure.” (Primary, Headteacher)

The advantage of testing at school noted by one secondary-school headteacher was that they would be properly conducted and not rely on parental adherence. However, home-testing was preferred to on-site and considered feasible to implement in secondary schools. We also found that participants who were initially more sceptical of the accuracy and value of asymptomatic testing, changed their opinion after they had been in-use for a while and “seen to work” through evidence of positive test results. Finally, schools that actively promoted LFTs to parents and students appeared to have higher uptake rates. Student narratives supported these findings.

“I’m personally not bothered, but a lot of people said they would rather do it at home because they don’t want people trying to help them with it or trying to look at them while they’re … just things like that.” (Secondary, Student)

A student who thought more people in their year-group would have taken-up LFTs if they were more actively promoted explained:

“I think it was, sort of, given out as more of an optional thing […] They sort of sent an email but that was it. It wasn’t very, like, pushed.” (Secondary, Student)

‘Capacity’ constraints that influenced collective action to implement preventive measures mainly arose from staff absence and burnout. Absence of teachers through illness or having to self-isolate after being in close contact with someone with Covid-19 created significant challenges for the school's ability to function and added to the pressure on remaining staff.

Another important aspect of schools’ capacity was their financial resources, which influenced their ability to deal with staffing issues. One secondary headteacher discussed how money had to be taken from financial reserves saved for other education-related projects to pay for supply teachers.

Problems with the availability of supply staff, and concerns among primary schools that bringing in supply teachers would introduce an additional infection risk into the school, meant senior leaders were often redeployed to cover classes and administration, adding to their workload.

For many headteachers, infection control was a full-time job in itself, extending to evenings and weekends, especially during peaks in incidence. Firstly, large workloads from managing Covid-19 in addition to core duties, secondly, uncertainty about the future course of the pandemic and thirdly, little opportunity for social support, led to stress and burnout over time for some headteachers and teachers. This in turn could undermine schools' ‘capacity’ and ‘potential’ for collective action. Headteachers referred to this period as the worst of their career, feeling inclined to quit their jobs and “literally dropping from the workload”. In primary schools, headteachers were often solely responsible for managing a majority of Covid-19 prevention, which took its toll on them. A headteacher who mentioned being resilient in the first interview, said the following during their second:

“I can tell you that I've been very, very, exhausted, very tired. I’ve not been well, as in like physically well, either. So I think it's had a significant impact on my wellbeing and I was saying earlier is, I'm very aware that there's only so much I can do, and there'll be a time when that will stop.” (Primary, Headteacher, 2^nd^ interview)

Capacity was important however. Where headteachers were supported by a team, their workloads were considered more manageable:

“Because this has been a team effort, people within the team have come up with answers that I wouldn't have thought of … So it's, it's one person can't do it all. It's about having that team together. And it's about just working to get that, as well as it can be for your local solution.” (Secondary, Headteacher)

Perceptions of insufficient acknowledgement by the government of teachers' efforts were mentioned as demoralising, and was noted to have contributed to teachers’ stress. A headteacher also noted that instances where the efforts of teachers had been acknowledged, had not seemed sincere:

“It’s a shame that they say how grateful they are for the teachers and schools doing what they’ve done over the pandemic, and then say, they’re going to reduce early retirement. Their actions don’t support what they’re actually saying.” (Primary, Headteacher)

### 3.4 Reflexive monitoring

All schools undertook formal and informal ‘reflexive monitoring’. As advised in the guidance, schools completed risk-assessments and regularly reviewed them, weekly or every other week. This constituted formal reflexive monitoring, in which the headteacher and a few senior leaders met to review implementation and make changes. A headteacher noted using their risk-assessment document as a way to communicate openly with all stakeholders.

“We review it regularly and we always make sure it’s shared with all staff. Even music teachers who are not employed by us. Our cleaners are not employed by us, but they work in our school. So everyone sees it. It’s shared with governors. It’s shared with the local authority and I also share it with all our unions. So that’s something we’ve had in place since we’ve had to do all of this last year.” (Primary, Headteacher)

Schools also conducted informal reflexing monitoring. These processes could enhance the ‘capability’ (workability) of the intervention. For example, a secondary-school headteacher explained that keeping students in the same room all day had restricted their curriculum too much and was a challenge given their small building. On reflection, they decided instead to allow students to move between classrooms but required face-coverings when doing so (before this became part of government guidance), strict sanitising, one-way systems and other measures to minimise risk.

Over time, many schools reported deviating from government guidance, often adopting a more cautious approach by going beyond what was required. When infection rates were high in their area, many schools ignored government guidance that suggested bubbles could be merged or face-coverings dropped. Other schools instituted face-covering requirements for staff and/or students before guidance recommended their use. A headteacher reflected that the agency displayed by school leadership in making decisions based on local risk-assessments was beneficial in gaining staff confidence and improving staff ‘potential’.

“I guess my approach changed a little bit. Normally I would wait for guidance … Whereas this term, I did what I felt was right for ensuring what was safe for our school … I made decisions around what we needed to do, and I think that probably has a positive effect on the staff, because they know that decisions will be made in relation to keeping them safe, rather than doing what we've been asked to." (Primary, Headteacher, 2^nd^ interview)

Integration of measures that did not interfere with school commitments to education or student wellbeing occurred over time as they were routinised and processes were normalised. Some of these measures, such as staggered lunch or break times, one-way systems and improved hand hygiene, were noted as likely to be sustained by some schools even after infection rates declined and restrictions lifted as they worked well for the school community.

Headteachers reflected on the importance of their ‘capacity’ and ‘potential’ for reflexive thinking and learning new skills as key for dealing with this emergency.

“All headteachers have had to respond to the change in the policies, the change in practice, I think, even if you’ve been experienced 20 years. I think, just because you’re constantly having to adapt and do things that you’ve never done before and not trained to do … To be an expert in close contact and testing … We’ve had to up-scale and you’ve got to learn and just continue to adapt.” (Secondary, Headteacher)

## 4 Discussion

### 4.1 Summary of key findings

Building on previous cross-sectional studies (Hommes et al., 2021; Lorenc et al., 2020; Sundaram et al., 2021), our QLR study provides new insights into how implementation of Covid-19 prevention in schools unfolded over time in a rapidly changing emergency epidemiological and policy context. In terms of ‘sense-making’, as defined in May's GTI, school leaders overcame challenges with the ‘capability’ (workability) of the guidance in order to understand what was required and develop workable local plans. This was promoted by the high ‘potential’ (positive attitudes) of these leaders and additional local ‘capacity’ in the form of support from trust operations managers, headteacher networks and/or local public-health teams. For teachers, students and parents, open communication was key for ‘sense-making’. A global survey by the OECD found that key to safe re-opening of schools was transparent and well-communicated guidance with local flexibility in implementation (OECD, 2021).

Measures such as handwashing, enhanced cleaning and one-way systems were sustained over time, while other potentially more effective interventions (e.g., physical distancing of desks or ventilation), which posed challenges with workability in some schools, were not always committed to over time. Inadequate support from national policy and financial resources may have hindered schools from overcoming these challenges implementing more effective measures (Gurdasani et al., 2022). In terms of GTI's construct of ‘cognitive participation’, staff were initially committed to implementing measures, such as teachers distancing from students and bubbles in secondary schools, despite these conflicting with schools' traditional mission to promote student learning and wellbeing. However, this commitment then fluctuated depending on changing perceptions of local risks. Previous cross-sectional research suggested that the feasibility of physical distancing was a key concern among US school staff (Pattison et al., 2021). Our findings suggest that distancing was feasible to implement in the short-term but not sustainable longer-term at least in the English policy context. These experiences may differ in other countries and contexts.

Collective action to implement and adhere to measures, for example wearing face-coverings, which was considered unworkable at the start, generally improved over time as they were routinised. This finding is consistent with Canadian research reporting that the reality of face-covering use was less problematic than that anticipated by students (Coelho et al., 2022). The fact that government guidance explicitly recommended avoiding face-covering use for students in classrooms in autumn term 2020 (Department for Education, 2020a) may have contributed to initial doubts about feasibility. Our findings suggest that face-coverings were more likely to be used when they were strongly emphasised by school management and in policy recommendations. Over time, many schools instituted face-covering requirements even when they were not recommended in government guidance. In terms of GTI's construct of ‘potential’, we found decreasing parental attitudinal support for preventive measures over time, consistent with other research (Yoshida-Montezuma et al., 2021).

Asymptomatic testing of students on-site in secondary schools posed a major ‘capacity’ challenge, but home-testing was considered feasible and additionally helped re-assure staff regarding occupational safety. Other research in UK schools has found that local trust in testing was influenced by school endorsement of them (Denford et al., 2022). Additionally, our findings suggest that perceptions of the reliability of LFTs improved over time and with use, and that active promotion of LFTs by school management improved uptake.

‘Capacity’ constraints arising from staff absence and accumulating staff burnout could also undermine collective action to implement measures. Stress among teachers during the Covid-19 pandemic has been well-documented (Kim et al., 2021; Pressley, 2021; Robinson et al., 2022). Leaders in our study reported not only vastly increased workloads and stress, but also being unable to fully “switch-off” during weekends or holidays due to the need for continual case monitoring and management that they had been made responsible for, as well as DfE's lack of timeliness in issuing guidance. They also reported inadequate support from and acknowledgement by the government.

Finally, in terms of GTI's concept of ‘reflexive monitoring’, schools undertook regular formal risk-assessments as well as informal reflections on implementation. These informed locally-relevant adaptations to control measures to improve their workability. Such reflexive thinking was both supported by and contributed to improved ‘sense-making’ and confidence among leaders over time. Headteachers and teachers in our study reported aiming to maintain some innovations and preventive measures initially instituted as a pandemic response, which were considered useful beyond the pandemic. Our findings also suggest that when leaders demonstrated flexibility to learn new skills, demonstrated agency in their decision-making, and when schools collaborated with other school stakeholders and local communities, they were able to utilise and strengthen these relationships to successfully deliver Covid-19 prevention.

The application of GTI in the Covid-19 pandemic context differed from its traditional application in planned interventions in non-emergency contexts in several ways. The intervention studied here consisted of many different measures implemented as part of an emergency and these changed rapidly over time in response to disease epidemiology and policy. Schools were also implementing measures with the knowledge that they were likely temporary so that long-term ‘normalisation’ as defined in the GTI was not a goal (Herlitz et al., 2020). Furthermore, the success of implementation could feed back to influence intervention ‘workability’, as well as local ‘capacity’ and ‘potential’ which consequently affected subsequent implementation. For example, implementation could generate staff burnout which eroded schools' ‘capacity’ and ‘collective action’. Implementation could also conflict with schools' educational goals, at some points reducing staff potential and cognitive participation.

### 4.2 Limitations

Teachers, parents and students who participated in interviews were recruited through headteachers at the school and may hence have been more likely to have had a positive opinion of school management and prevention measures. While a majority undertook a second interview facilitating exploration of how accounts developed over time, this was not possible for all participants, especially students. Although data from all participants were analysed, this paper largely reports data from headteachers as they were most informative in explaining implementation processes. Interviews with other school staff and local public-health teams, who may have had additional perspectives, were not conducted due to feasibility constraints. Our findings suggest they were not central to school implementation of preventive measures, which was the focus of the study. A total of 74 interviews were conducted with 43 participants, which is generally considered a very large sample for qualitative longitudinal research. However, additional interviews may have revealed further insights. It is unlikely that participants who were not fluent in English were interviewed, as all interview were conducted in English. We also did not collect information on participant socio-economic status or migrant status. Finally, we were unable to conduct direct observations and interviews were conducted over the telephone, due to Covid-restrictions. However, we found that this did not necessarily impact rapport-building or depth of conversations, and telephone interviews allowed us to reach otherwise busy participants at their convenience.

### 4.3 Implications for policy and research

Our study suggests that it was possible for schools to implement and sustain a range of preventive measures. Successful implementation of preventive measures may be one explanation for why there is little evidence that reopening schools was associated with major increases in community infections (Walsh et al., 2021). Some measures, such as face-coverings and testing, were found to be feasible despite initially being viewed in schools as unfeasible. Others, notably physical distancing in primary and the use of ‘bubbles’ in secondary schools were not considered sustainable in the long-term.

The value of open and responsive communication by school leaders in gaining staff trust, cooperation with other stakeholders, and therefore better implementation, underscores the importance of effective communication in a crisis.

Implementation was also supported when schools could call on assistance from school and teacher networks, local authorities and public-health teams. Schools that were a part of multi-school trusts or academies benefited from additional support offered by operations managers who handled interpretation of guidance and risk assessments. Pooling of resources to support some school functions in any future pandemic responses or for pandemic preparedness should therefore be considered. Regular internal review processes were an effective means of maintaining and adapting implementation and were a valuable form of communication with stakeholders.

Our findings suggest that protecting school staff wellbeing and acknowledging their efforts should be paramount during an emergency to minimise burnout and maintain morale. Disengaging from work outside of work hours and social appreciation has previously been found to reduce workplace stress (Sonnentag, 2012; Upadyaya et al., 2021). Particular attention to gender may also be warranted as three-quarters of teachers in England are women (Department for Education, 2020b) and evidence suggests that female teachers had increased work-related anxiety than men during the pandemic (John Jerrim - FFT Education Datalab, 2022). Prioritising staff wellbeing could help promote school potential for implementation and may result in greater sustainment of prevention measures (Sundaram et al., 2022).

In terms of research, we conclude that the GTI is a useful framework for future studies of implementation of preventive measures in pandemics or other emergencies. The GTI may require adaptation when used to examine implementation in emergency contexts to take into account changing and sometimes contradictory objectives, time-varying factors and feedback loops from implementation impacting intervention embeddedness.

## Funding

This work was supported by the Department of Health and Social Care, United Kingdom. The funder had no role in the study design, data collection, data analysis and interpretation, writing or the decision to submit this article for publication. The authors alone are responsible for the views expressed in this publication and they do not necessarily represent the decisions, policy or views of the Department for Health and Social Care, the Office for National Statistics or the UK Health Security Agency. SML was supported by a Wellcome Trust Senior Research Fellowship in Clinical Science (205039/Z/16/Z). This research was funded in whole or in part by the Wellcome Trust [205039/Z/16/Z]. For the purpose of Open Access, the author has applied a CC BY public copyright licence to any Author Accepted Manuscript (AAM) version arising from this submission.

## Author contributions

**NS:** Conceptualisation and design, Data collection, Analysis, Writing – Original draft, review and editing, Project administration; **NT and LC**: Data collection, Analysis, Writing – Review and editing; **PH:** Data collection, Writing – Review; **PND**: Conceptualisation, Writing – Review and editing; **SML and JH**: Conceptualisation and design, Writing – Review and editing, Funding acquisition; **CB:** Conceptualisation and design, Analysis, Writing – Review and editing, Funding acquisition, Supervision. All authors have read and approved the final manuscript.

## Declaration of competing interest

The authors declare that they have no known competing financial interests or personal relationships that could have appeared to influence the work reported in this paper.
